# Synfire chains and gamma oscillations: two complementary modes of information transmission in cortical networks

**DOI:** 10.1186/1471-2202-14-S1-P226

**Published:** 2013-07-08

**Authors:** Gerald Hahn, Alejandro F Bujan, Yves Fregnac, Ad Aertsen, Arvind Kumar

**Affiliations:** 1 Unité de Neuroscience, Information et Complexité (U.N.I.C), CNRS, Gif sur Yvette, UPR 3293, France; 2 Bernstein Center Freiburg, Neurobiology & Biophysics, Faculty of Biology, University of Freiburg, Freiburg im Breisgau, 79014, Germany

## Background

The cortex is thought to process sensory stimuli from the environment by flexible routing of neuronal activity across a hierarchy of functionally specialized neuronal networks. This routing necessitates mechanisms that allow for high fidelity communication of neuronal activity between these networks [1]. It was suggested that synchronization of spiking activity plays a pivotal role in this communication process, based on which two seemingly different mechanisms were proposed. The synfire chain hypothesis postulates the existence of highly organized divergent/convergent connections, which allow the generation and faithful transmission of synchronous spike volleys generated by common drive from presynaptic neurons [2]. By contrast, another model proposes that communication between different brain areas is achieved by creating consistent phase relations between population level oscillations entrained by distinct neuronal networks. These oscillations emerge as a consequence of local interactions between excitatory and inhibitory neurons. So far, synchronization driven by oscillations and synchronization due to a common drive have been considered as dynamical processes of a different nature. Here, we outline a new theoretical framework, which views the appearance of coherent oscillations as a manifestation of common input synchrony spreading along diluted feed-forward networks (FFNs), which, initially, fail to create stable propagation of excitatory spike volleys due to insufficient weight and number of connections. We have tested this working hypothesis by implementing numerical simulations of diluted FFNs. In our network model, each FFN group consisted of recurrently connected leaky integrate-and fire neurons with an excitation-inhibition ratio of 4:1.

## Results

In our simulations, external stimulation with rhythmic pulse packets was followed by network activity oscillations, which were a consequence of mutual interactions between the excitatory and inhibitory pools. These oscillations progressively amplified in strength with each new input presentation. They synchronized excitatory activity in each FFN pool and facilitated the propagation of excitatory spike volleys along weak and sparse divergent/convergent connections. Several oscillation cycles were needed to transmit spike volleys across the entire FFN in contrast to synfire activity, in which excitation is propagated in one sweep. We also hypothesized that the precise timing inherent to coherent oscillations may induce synaptic potentiation, which would reduce the number of oscillation cycles necessary to propagate synchrony and drive the network towards synfire chain dynamics. Indeed, our simulations confirmed that an increase of synaptic weights between groups of the FFN transformed oscillation chains into classical 'synfire chains', in which synchrony was transmitted in a single wave. In summary, we propose a conceptual link between the concepts of synfire chains, coherent oscillations and synaptic plasticity. We suggest that coherent oscillatory dynamics presents an immature case of spike volley transmission across multiple neuronal networks, which may lead to secured transmission, without the need for oscillations, via the results of synaptic plasticity.
